# Supplementary material for: Band conductivity oscillations in a gate-tunable graphene superlattice
Source: Nat Commun. 2022 May 23;13:2856. doi: 10.1038/s41467-022-30334-3 (PMC9126977; doi:10.1038/s41467-022-30334-3)
Supplement: Supplementary file 1 — Supplementary Information [file 41467_2022_30334_MOESM1_ESM.pdf]

Band conductivity oscillations in a gate-tunable graphene superlattice – Supplementary Information

Robin Huber,[1] Max-Niklas Steffen,[2] Martin Drienovsky,[1] Andreas Sandner,[1] Kenji Watanabe,[3] Takashi Taniguchi,[4] Daniela Pfannkuche,[2] Dieter Weiss,[1] and Jonathan Eroms[1,*]

[1]*Institute of Experimental and Applied Physics, University of Regensburg, D-93040 Regensburg, Germany*
[2]*I. Institute of Theoretical Physics, University of Hamburg, Notkestraße 9–11, D-22607 Hamburg, Germany*
[3]*Research Center for Functional Materials, National Institute for Materials Science, 1-1 Namiki, Tsukuba 305-0044, Japan*
[4]*International Center for Materials Nanoarchitectonics, National Institute for Materials Science, 1-1 Namiki, Tsukuba 305-0044, Japan*

(Dated: February 18, 2022)

I. LOW-TEMPERATURE CHARACTERIZATION AND MAGNETOTRANSPORT

The sample described in the main text was employed for low-temperature measurements featuring miniband structure, Landau fan signatures of the Hofstadter butterfly and non-monotonic sequence of quantum Hall plateaus, which were reported previously [1]. For convenience, we reproduce the first three figures from the journal publication, see Figs. S1, S2 and S3. The satellite Dirac peaks, visible in the experiment (Fig. S1) at high back gate voltages, can be identified by comparison to transport and band structure calculations in Fig. S2.

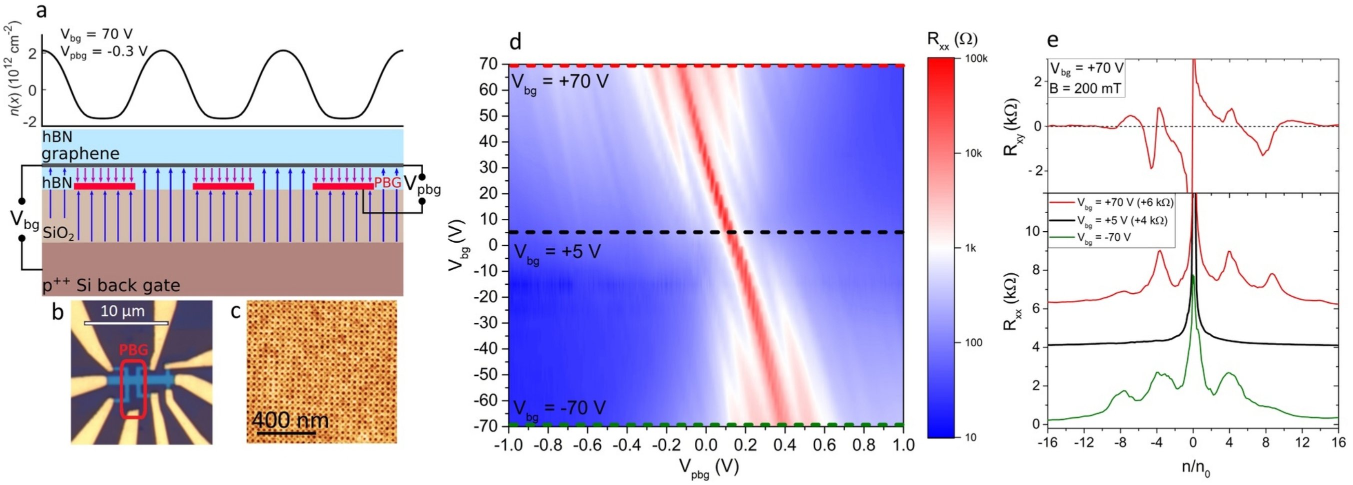

FIG. S1. **Sample Layout and Gate Response at Zero Magnetic Field.** **a** Schematic of the sample geometry. Due to the interplay between a Si back gate and a few-layer graphene patterned bottom gate (PBG), a periodic charge carrier density modulation can be induced in the encapsulated graphene layer on top of the two gates. **b** Micrograph of the studied device in Hall bar geometry and Cr/Au edge contacts. Red line marking the position of the PBG. **c** AFM picture of the PBG with a square superlattice and a lattice constant of $a = 40$ nm. For the studied sample, a periodic array of holes was etched into a bilayer graphene flake. **d** Gate map of the device at $T = 1.5$ K. Longitudinal resistance R_{xx} as a function of back gate voltage V_{bg} and PBG voltage V_{pbg}. By increasing the back gate voltage, *i.e.*, increasing the modulation strength, satellite peaks start to occur besides the main Dirac peak. **e** Linecuts at three different back gate voltages highlight the additional features upon tuning the periodic potential strength. Upper panel shows the sign change of the corresponding Hall resistance R_{xy} at the position of the satellite Dirac points at $B = 200$ mT. Reprinted with permission from [1], Copyright 2020: American Chemical Society.

* jonathan.eroms@ur.de

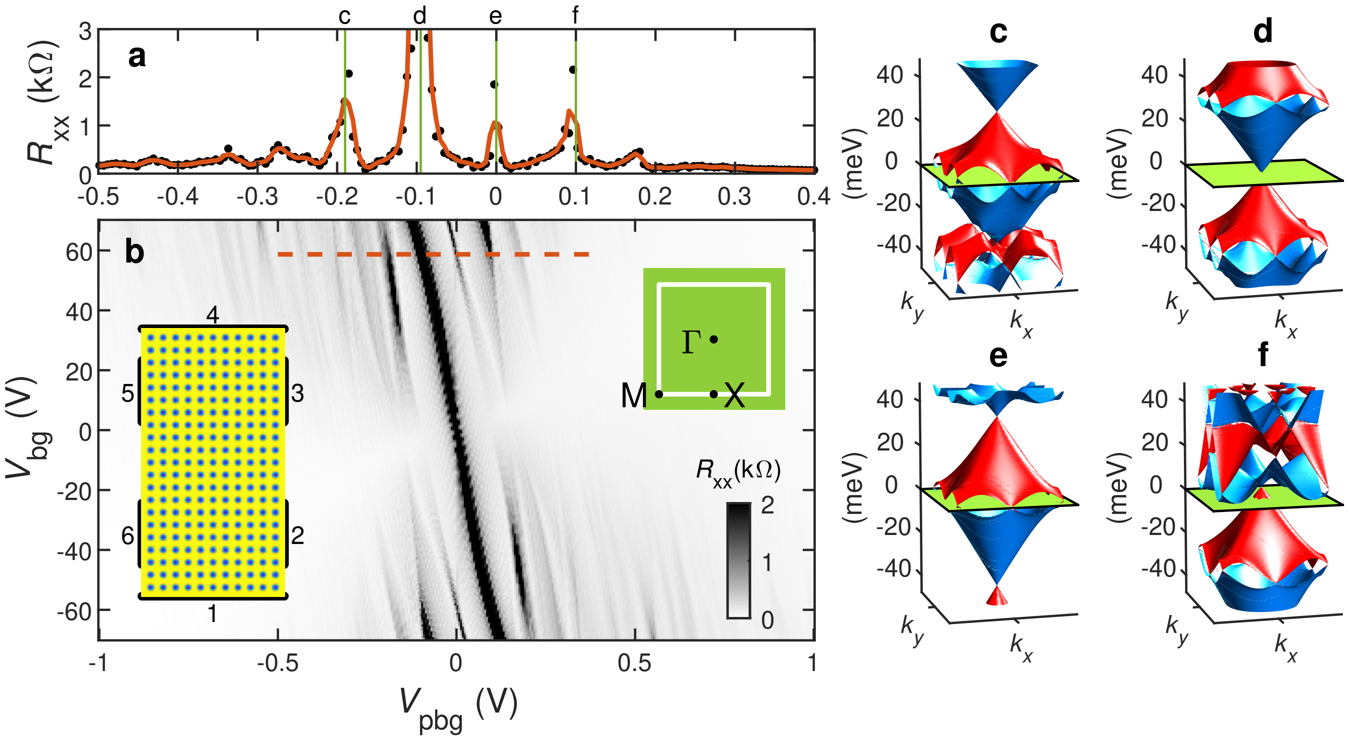

FIG. S2. **Transport Simulation and Band Structure.** **a** Four-terminal longitudinal resistance R_{xx} taken at $V_{\mathrm{bg}} = 58$ V (dashed line in **b**). Continuous line shows smoothed data, and the black dots are the raw simulation results. Marked positions correspond to band structure plots. **b** $R_{xx}(V_{\mathrm{pbg}}, V_{\mathrm{bg}})$ at zero magnetic field. Left inset: Geometry of the simulation. Right inset: Mini-Brillouin zone of the square superlattice with points of high symmetry. **c** to **f**: Band structure plots at positions marked in **a**. In each diagram, the green plane shows the position of the Fermi level. Reprinted with permission from [1], Copyright 2020: American Chemical Society.

II. WEISS OSCILLATIONS AND BROWN-ZAK OSCILLATIONS AT LARGE BACK GATE VOLTAGES

Figure S4 contains the data of Fig. 4ab of the main text at different contrast setting to better show the modulation of BZ visibility by WOs and data from a separate run. In the unipolar regime, graphene regions influenced mainly by back gate, or patterned bottom gate, respectively, are populated by the same carrier type, and thus the modulation potential is weaker than in the bipolar regime. In the unipolar regime, the limit of weak modulation potential is realized, as in the case for low back gate voltage (Fig 4c of the main text) for the entire PBG voltage range. Therefore, at the flat band condition, the BZ feature (vertical line) at $\phi/\phi_0 = 1$ is broken, and the feature at $\phi/\phi_0 = 2$ is fully suppressed. For $V_{bg} = -80$ V, the latter observation is better made at the gray scale setting of Fig 4b in the main text.

III. BROWN-ZAK OSCILLATIONS IN AN ARTIFICIAL SUPERLATTICE IN COMBINATION WITH A MOIRÉ SUPERLATTICE

Figure S5a displays a plot of d^2G/dB^2 as a function of magnetic field B and PBG voltage V_{pbg} at $V_{bg} = 100$ V measured at a temperature of $T = 125$ K for a sample with an artificial hexagonal superlattice (with $a = 40$ nm) in combination with a moiré superlattice. In the vicinity of the moiré peaks, pronounced Brown-Zak oscillations occur which reflect the periodicity of the moiré superstructure. At lower charge carrier densities, i.e. in a region close to the main CNP (around $V_{pbg} \sim 0$ V), weaker features of enhanced band conductivity start to appear stemming from the artificial superlattice. Figure S5b shows data at $V_{pbg} = 1.2$ V in a region dominated by Brown-Zak oscillations of the moiré superstructure. By evaluating the peak positions of the most pronounced peaks (corresponding to unit fractions of ϕ/ϕ_0 per superlattice unit cell area $A = \sqrt{3}a^2/2$), a lattice constant of $a \sim 14$ nm can be estimated. Figure S5c displays R_{xx} as a function magnetic field B at $V_{pbg} = 0.4$ V. Brown-Zak features due to the artificial hexagonal superlattice are visible with $\phi/\phi_0 = 1$ at about $B_0 \sim 3$ T. In this case, a lattice constant of about $a \sim 40$ nm can be

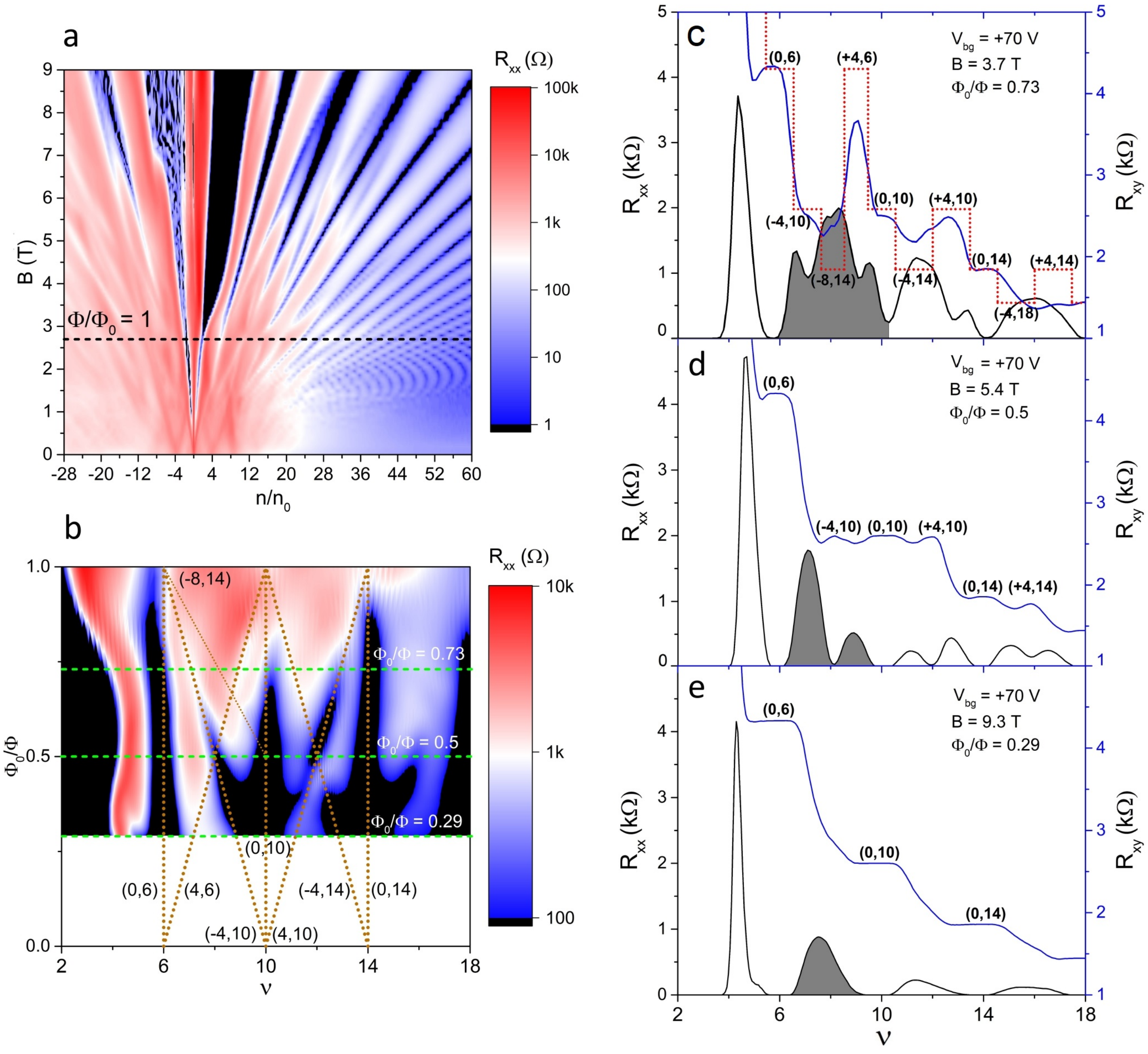

FIG. S3. **Experimental Magnetotransport Data at Large Fields.** **a** R_{xx} plotted as a function of magnetic field and normalised charge carrier density n/n_0. Landau fans emerge from the main Dirac point and satellite Dirac points giving rise to superlattice induced energy gaps manifesting as additional minima in longitudinal resistance. The data were taken at $T = 1.5$ K and a back gate voltage of $V_{\mathrm{bg}} = 70$ V. **b** Replot of the data in **a** in a Wannier diagram with Landau level filling factor ν and inverse magnetic flux ϕ_0/ϕ. Vertical minima correspond to energy gaps between Landau levels, additional diagonal features are signatures of the largest energy gaps of the Hofstadter butterfly energy spectrum. Dotted lines highlight the Landau gaps (vertical) and the most pronounced Hofstadter gaps (diagonal) labeled by (s,t) in the second and third Landau level. **c** to **e** show linecuts at certain magnetic fields from the data in **b** showing the evolution of the magnetic bandstructure upon changing the magnetic flux per superlattice unit cell area. Energy gaps in the spectrum correspond to minima in R_{xx} and plateaus in R_{xy} and follow the equation $\nu = (\phi_0/\phi)s + t$. The observed energy gaps are labeled by their parameters (s, t). Features with $s \neq 0$ correspond to superlattice induced energy gaps. The dotted red line in **c** shows the ideal non-monotonic quantum Hall sequence evaluated for $s = 0, \pm 4$ and an additional energy gap with $(s, t) = (-8, 14)$. The grey-shaded area in the linecuts highlights the evolution of the second Landau level as a function of inverse magnetic flux. Reprinted with permission from [1], Copyright 2020: American Chemical Society.

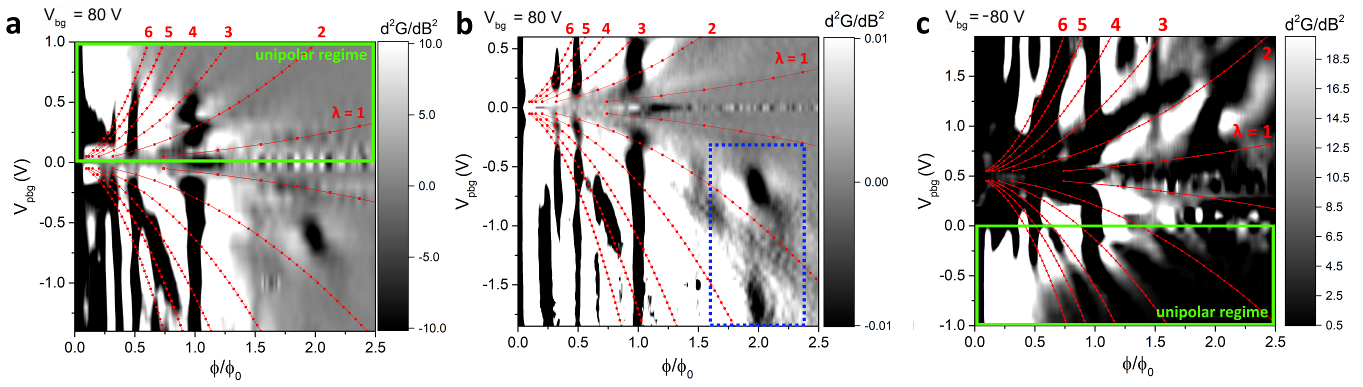

FIG. S4. Gray scale plots of d^2G/dB^2 for $V_{bg} = \pm 80$ V. Data in panel **a** were taken in a separate run, data in **b** and **c** are the same as in Fig. 4ab of the main text, but with different contrast setting. In the unipolar regime (outlined in green), the modulation potential is weak, and the BZ feature at $\phi/\phi_0 = 1$ is suppressed at the flat band condition of the WOs (red lines). This is particularly well visible at $V_{bg} = +80V$ (panel **a**). The BZ feature at $\phi/\phi_0 = 2$ is even fully suppressed at the flat band conditions (panel **b**, dashed blue box).

estimated which is in good agreement with the designed lattice period of the artificial hexagonal superlattice.

IV. DEPENDENCE OF THE VISIBILITY OF THE HOFSTADTER FINE STRUCTURE ON THE LANDAU LEVEL BAND WIDTH

Figure S6 illustrates the dependence of the visibility of the Hofstadter fine structure on the Landau band width. Figure S6a (see also Fig. S3) shows low-temperature magnetotransport data of our previous work [1]. R_{xx} is plotted as a function of inverse magnetic flux ϕ_0/ϕ and Landau level filling factor ν. Energy gaps (corresponding to minima in R_{xx}) follow the Diophantine equation, [2–4]

$$\nu = (\phi_0/\phi)s + t, \tag{1}$$

where s and t are integer parameters. Manifestations of energy gaps in the magnetotransport data are labeled with their integer parameters (s, t) (dotted lines). The vertical minima correspond to Landau gaps of pristine graphene (with $s = 0$), the diagonal minima correspond to Hofstadter minigaps (with $s \neq 0$). Figure S6b displays the calculated Landau band width $|\Delta E_n|$ (see Eq. (3) in the main text) in units of the modulation potential amplitude V_0. At large Landau band width, the Hofstadter related features in the magnetotransport data are best developed and visible, since the minigaps in the spectrum in the most extended Landau bands obtain their largest magnitude. At small Landau band width, the internal structure of the Landau levels can not be resolved.

V. CALCULATED MODULATION OF BROWN-ZAK FEATURES BY OCCURRENCE OF FLAT BANDS

Figure S7 depicts the calculated band conductivity contribution as a function of magnetic flux ϕ/ϕ_0 and charge carrier density n_s, shown as a three-dimensional plot. The band conductivity features at $\phi/\phi_0 = 1$ and $\phi/\phi_0 = 2$ are visibly modulated by the occurrence of flat bands in the spectrum. At smaller values of ϕ/ϕ_0, the effect of modulation is mainly suppressed due to thermal smearing.

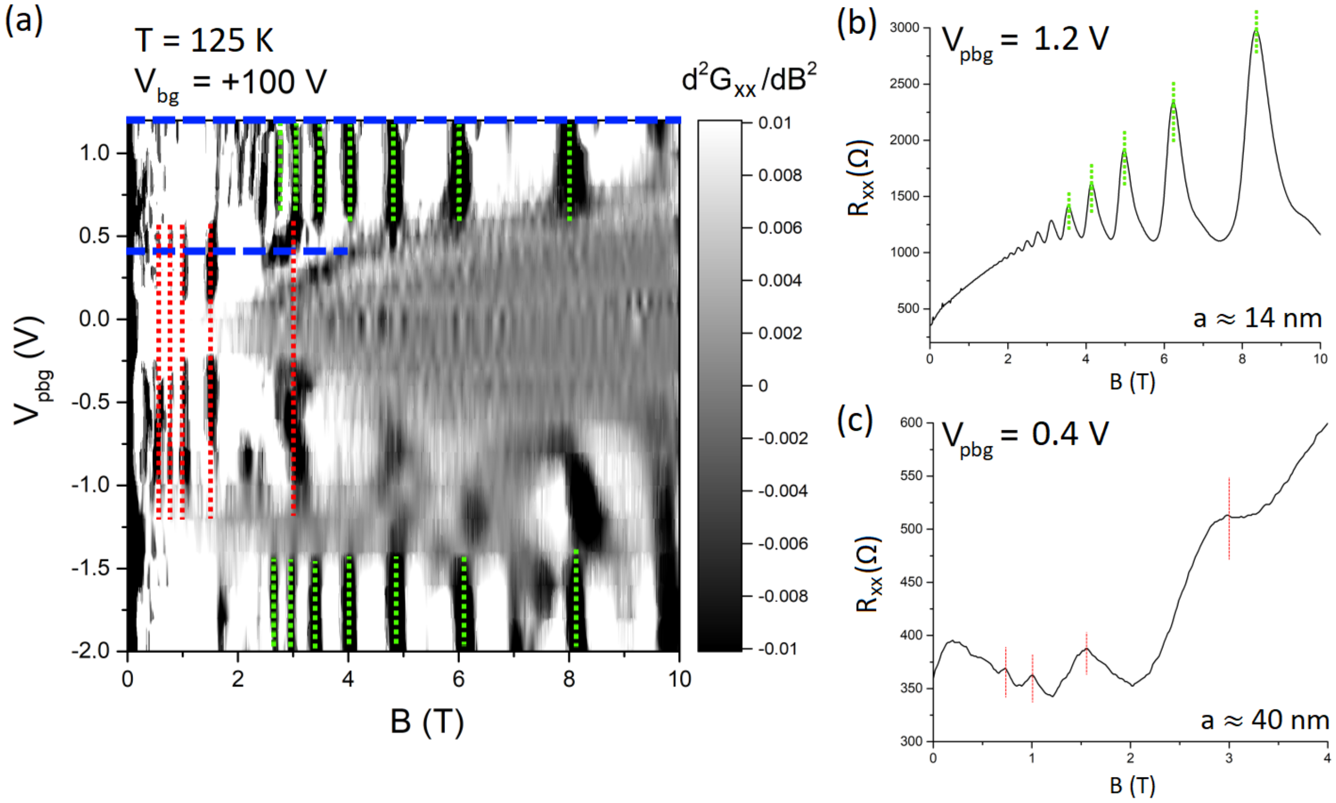

FIG. S5. **(a)** d^2G_{xx}/dB^2 as a function of magnetic field B and PBG voltage V_{pbg} at $V_{bg} = 100$ V measured at a temperature of $T = 125$ K. Brown-Zak oscillations due to a moiré superlattice (green dotted lines) and Brown-Zak oscillations induced due to a gate-defined superlattice are visible (red dotted lines). **(b)** R_{xx} plotted as a function of magnetic field B at $V_{pbg} = 1.2$ V corresponding to the upper blue dashed line in (a). A moiré lattice constant of $a \sim 14$ nm can be estimated by evaluating the peak positions (green dotted lines). **(c)** R_{xx} plotted as a function of magnetic field B at $V_{pbg} = 0.4$ V corresponding to the lower blue dashed line in (a). Brown-Zak oscillations, induced by the artificial superlattice, are visible with $\Phi/\Phi_0 = 1$ at about $B_0 \sim 3$ T. A lattice constant of $a \sim 40$ nm can be estimated by evaluating the peak positions (red dotted lines) which is in good agreement with the artificially designed hexagonal superlattice.

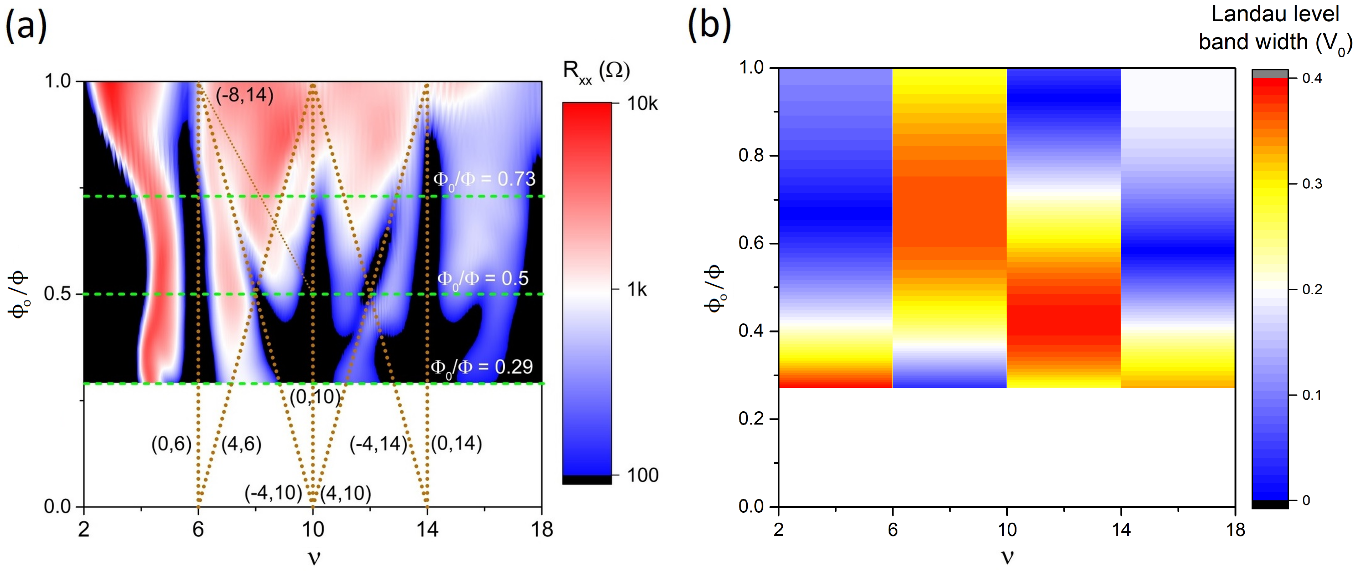

FIG. S6. **(a)** Magnetotransport data at $T = 1.5$ K. R_{xx} is plotted as a function of inverse magnetic flux ϕ_0/ϕ and Landau level filling factor ν [1]. **(b)** Calculated Landau band width $|\Delta E_n|$ in units of the modulation potential amplitude V_0.

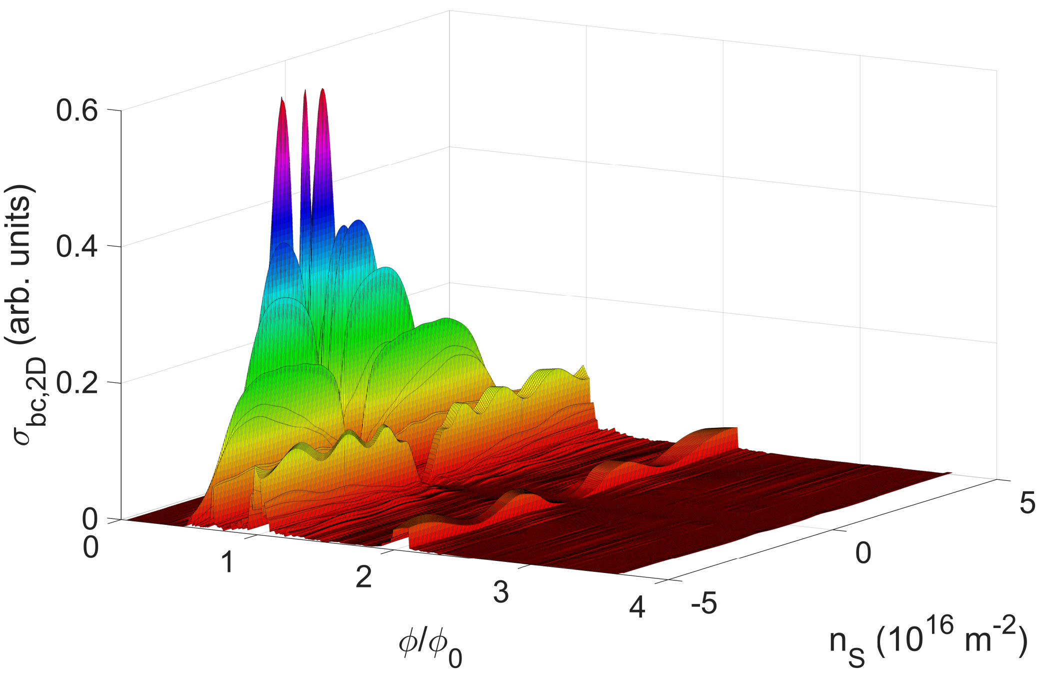

FIG. S7. 3D plot of the band conductivity contribution as a function of magnetic flux ϕ/ϕ_0 and charge carrier density n_s. The features at $\phi/\phi_0 = 1$ and $\phi/\phi_0 = 2$ are visibly modulated by the occurrence of flat bands in the spectrum.

[1] R. Huber, M.-H. Liu, S.-C. Chen, M. Drienovsky, A. Sandner, K. Watanabe, T. Taniguchi, K. Richter, D. Weiss and J. Eroms. *Gate-Tunable Two-Dimensional Superlattices in Graphene.* Nano Letters **20**, 8046 (2020).

[2] G. H. Wannier. *A Result Not Dependent on Rationality for Bloch Electrons in a Magnetic Field.* physica status solidi (b) **88**, 757 (1978).

[3] D. J. Thouless, M. Kohmoto, M. P. Nightingale and M. den Nijs. *Quantized Hall Conductance in a Two-Dimensional Periodic Potential.* Phys. Rev. Lett. **49**, 405 (1982).

[4] A. H. MacDonald. *Landau-level subband structure of electrons on a square lattice.* Phys. Rev. B **28**, 6713 (1983).
